# Intestinal Microbiota as Modulators of the Immune System

**DOI:** 10.1155/2015/159094

**Published:** 2015-03-02

**Authors:** Borja Sánchez, Miguel Gueimonde, Amado Salvador Peña, David Bernardo

**Affiliations:** ^1^Nutrition and Bromatology Group, Department of Analytical and Food Chemistry, Food Science and Technology Faculty, University of Vigo, Ourense Campus, 32004 Ourense, Spain; ^2^Department of Microbiology and Biochemistry of Dairy Products, Instituto de Productos Lácteos de Asturias (IPLA-CSIC), 33300 Villaviciosa, Spain; ^3^Laboratory of Immunogenetics, Department of Medical Microbiology and Infection Control, VU University Medical Centre, P.O. Box 7057, 1007 MB Amsterdam, Netherlands; ^4^Antigen Presentation Research Group, Imperial College London, Northwick Park and St. Mark's Campus, Level 7W St. Mark's Hospital, Watford Road, Harrow HA1 3UJ, UK

The gastrointestinal immune system is exposed to a large amount of different products mainly innocuous (derived from “friendly” bacteria and/or food antigens) but sometimes also dangerous and infectious (as invading bacteria or viruses). Despite that, it is effective in discriminating between them and hence maintaining immune tolerance against the natural inhabitants of our gut, but initiating immune responses against the harmful invading microorganisms [1, 2]. In the last decades, the western lifestyle has seen an increase in the prevalence of immunoregulatory disorders which has been linked to changes in the microbiota composition due to the increased use of antibiotics and the absence of intestinal parasites as proposed in the “hygiene hypothesis.” Indeed, the immune system has become more dependent upon the microbiota and the natural environment [3]. However, recent data indicate that helminth-induced immunomodulation occurs independently of changes in the microbiota [4].

The commensal microbiota plays a central role modulating the outcome of immune responses in the gastrointestinal tract keeping immune homeostasis in health [5]. Indeed, germ-free animals have an immature immune system and can develop inflammation which is reverted once the microbiota is conventionalized [6]. The commensal microbiota also has the capacity to modulate several aspects of the host including its physiology and/or nutritional status contributing therefore to several diseases affecting not only the gut but also distant organs [7–9]. Therefore, not surprisingly the gut microbiota modulation (via pre/probiotics or through faecal microbiota transplants) appears a very promising area of research aiming to modulate the outcome of the immune system looking for an impact in the clinics. In this context, several clinical trials are underway to assess the true efficacy of faecal microbiota transplantations [10] as well as their long term effects [11, 12]. In agreement with that, there are still many factors regarding the host/microbiota cross talk which remain obscure and that have to be addressed to further our understanding about how the microbiota can modulate the outcome of the immune responses in the host.

In this special issue, we have therefore aimed to gain depth into the current understanding of immune processes in the human gastrointestinal tract in health and disease by selecting work in progress of active investigators in the field.

The study by C. M. C. Maranduba et al. reviews the most recent advances on intestinal microbiota and its role in the maintenance of the host homeostasis. The review describes, in a detailed manner, the interaction of the microbiota with the intestinal immune system and the mechanisms involved in such interaction. The authors also discuss the latest results on a current hot topic in microbiota research: the interaction with the nervous system and the impact upon the gut-brain axis [13]. This research area is attracting increasing attention for elucidating the impact of the microbiota beyond the classically studied intestinal interactions, which promises to expand our understanding on the role of the microbiota on human biology in broad terms.

In a similar context, C. Ferreira et al. discuss the impact of microbes on the gut-associated immune system function and, moreover, on the onset and development of inflammatory disorders. An exhaustive revision of metagenomic and animal data in the framework of different diseases, mainly inflammatory bowel disease, asthma, and obesity, showed deep alteration on the gut-associated microbiota profiles, as well as deficiencies in the immune response. Alterations in the intestinal microbiota composition promote systemic inflammation that is a hallmark of obesity and subsequent insulin resistance [14].

Pre- and probiotics have been extensively used for improving the balance of the intestinal microbiota and immune response modulation [15, 16]. A human target group that may benefit very much from strategies aimed at the modulation of the gut microbiota and the stimulation of the immune system is that of premature newborns. It is known that in these infants both the microbiota establishment process and the immunity are altered. In this issue L. Moles et al. report the results of a pilot study on the effects of the administration of two probiotic strains, isolated from human milk, to preterm infants. The authors evaluated several microbiological and immunological markers observing the ability of the strains to modulate the microbiota and to survive the gastrointestinal passage. Moreover, a reduction of fecal calprotectin, an inflammatory marker, was observed throughout the probiotic treatment in agreement with previous observations [17, 18].

Continuing with the relevance of probiotics, P. Carasi et al. administered the strain* Lactobacillus kefiri* CIDCA 8348 to healthy mice during 21 days. This strain was chosen in a previous study for its ability to induce chemokine CCL20 gene expression, an attractant of immune cells. The overall impact of* L. kefiri* on the mouse gut-associated immune system varied from an increase in fecal IgA to the reduction of several proinflammatory mediators in Peyer patches. Overexpression of interleukin 10 and mucin 6 genes in the ileum and the ability of* L. kefiri* to reduce the proinflammatory effects of lipopolysaccharide make strain CIDCA 8348 a candidate to be included in functional foods targeting inflammatory bowel disorders.

We have also selected manuscripts which discuss the role of the microbiota not only in immune homeostasis, but also in different diseases like HIV and* Helicobacter pylori* infection. The gastrointestinal tract has been recently described as the main HIV reservoir in the human body. While in healthy controls there is a reciprocal cross talk between the commensal microbiota and the host, HIV infection can dramatically affect both the microbiome and the host's immune system adding therefore a third factor to the dialogue in these patients. In this special issue, K. Vyboh et al. have reviewed the impact of HIV infection in the gastrointestinal immune system and how that can lead to changes not only in the microbiota composition and function, but also in the mucosal permeability resulting in microbial translocation from the lumen. As a consequence, viral and microbial factors work together in the patients creating a positive feedback mechanism which enhances HIV progression leading to a vicious cycle of immune activation [19, 20].

*Helicobacter pylori* infection is one of the most common causes of chronic gastritis. In this issue, L. A. Cherdantseva et al. performed a histological examination of the gastric mucosa during development of chronic gastritis in these patients. Their findings confirmed that* H. pylori* infection causes an increase in the number of infiltrating immune cells, including macrophages and lymphocytes which also had an enhanced capacity to secrete proinflammatory nitric oxide synthase which may allow an accumulation of free radicals in the tissues leading to an aggravation of the inflammatory process with impaired regeneration processes.

Moving towards more immunologically related studies, the role of intestinal dendritic cells (DCs) in the gastrointestinal compartment cannot be avoided as they are specialized antigen-presenting cells with the ability to extend their dendrites between epithelial cells and directly sample bacteria from the intestinal content [21, 22]. In a former study, M. Wiese et al. [23] selected* Lactobacillus* strains active against* H. pylori*. In their new research, the authors used monocyte-derived DCs for assessing the immunomodulatory abilities of those previously selected strains in the presence or absence of* Helicobacter pylori*. Both lactobacilli species were able to increase the maturation of DCs and to induce the production of IL23. However, the strains differed in their ability to induce IL-10 leading to different IL-10/IL-12p70 ratios. Altogether, the results presented suggest that the* H. pylori*-induced DCs tolerogenic phenotype may be overcome by the presence of certain lactobacilli.

Regulatory T-cells (Tregs) are essential to maintain immune homeostasis as they are critical for prevention of spontaneous inflammation. While development of Tregs requires the presence of TGF*β* at the time of the antigen presentation elicited by DCs, the presence of IL-6 promotes T-cell differentiation towards a Th17 proinflammatory profile as seen in several autoimmune diseases including rheumatoid arthritis (RA). In their review, R. Rogier et al. discuss the mechanisms by which the intestinal microbiota can influence the Th balance in the lamina propria. To that end, the authors have reviewed the background information about RA being a Th17 disease, how the intestinal microbiota can modulate the outcome of immune responses, and the evidence linking, in both in vivo and animal models, the commensal microbiota with RA development likely via TLR recognition by the host.

Continuing with Tregs, information regarding their development in the neonatal liver is scarce. In their study, A. Maria et al. describe how Treg can be already found on the third day after birth in the murine thymus, spleen, and liver. However, by the first week of life the frequency of liver Treg cells exceeds that of the spleen by 1.5–2-fold in a transient manner since 6 weeks after birth frequency of liver Tregs was reduced. Given that conventionalization of germ-free animals usually leads to a rapid expansion and mucosal Tregs, and considering that the liver receives most of its blood flow via the intestinal portal vein, the authors hypothesized and proved that the transient increased in neonatal liver Tregs was controlled by the intestinal microbiota as differences between frequency of liver and spleen Treg were abrogated in MyD88 knockout animals. This study expands our current knowledge on how the intestinal microbiota can also modulate the immune properties of tissues where they do not get direct access and also about the mechanisms of liver tolerance development.

As stated before, the intestinal immune system and the beneficial microorganisms within the lumen of the intestinal host communicate extensively to eliminate pathogens and markers to activate the innate and acquired immune response are necessary. In this issue, K. Radulovic and J. H. Niess review the role of CD69 which is highly expressed in intestinal T-cells. They propose that not only is this molecule just an activation marker but also it is essential in the regulation of intestinal inflammation. They review the evidence about how microbial-derived factors recognized by pattern recognition receptors could contribute to the CD69 expression on the surface of colonic T-cells and may be involved in lymphocyte migration in particular in inflammatory bowel disease. Although the authors are fully aware that most of the data come from mice research, they propose that since the intestinal microflora also regulates this marker in intestinal inflammation it may be a good target molecule for the treatment of inflammatory bowel disease.

Finally, an excellent review by M. J. B. Silva et al. covering many of the aspects described earlier in this editorial and the mechanisms involved in the modulation of host-microbe interactions has also been selected. It summarizes the possible effects of the breakdown of the homeostatic association that can lead to intestinal inflammation and pathology.

It has been a pleasure to select the work presented in these areas by experts in the respective fields. We hope that their findings will help to enrich the knowledge of the mediators of inflammation of the human gastrointestinal tract and will form the basis for new approaches to the treatment of common infections and those conditions that although rare have such a bad prognosis.

*Borja Sánchez* *Borja Sánchez* *Miguel Gueimonde* *Miguel Gueimonde* *Amado Salvador Peña* *Amado Salvador Peña* *David Bernardo* *David Bernardo*
